# Precision Oncology, Artificial Intelligence, and Novel Therapeutic Advancements in the Diagnosis, Prevention, and Treatment of Cancer: Highlights from the 59th Irish Association for Cancer Research (IACR) Annual Conference

**DOI:** 10.3390/cancers16111989

**Published:** 2024-05-23

**Authors:** Seodhna M. Lynch, Aisling B. Heeran, Caoimbhe Burke, Niamh Lynam-Lennon, Alex J. Eustace, Kellie Dean, Tracy Robson, Arman Rahman, Simone Marcone

**Affiliations:** 1 Personalised Medicine Centre, School of Medicine, Ulster University, C-TRIC Building, Altnagelvin Area Hospital, Glenshane Road, Londonderry BT47 6SB, UK; s.lynch1@ulster.ac.uk; 2 Department of Surgery, Trinity Translational Medicine Institute, Trinity St. James’s Cancer Institute, Trinity College Dublin, D02 PN40 Dublin, Ireland; heerana@tcd.ie (A.B.H.); lynamlen@tcd.ie (N.L.-L.); marcones@tcd.ie (S.M.); 3 UCD School of Biomolecular and Biomedical Science, UCD Conway Institute, University College Dublin, Belfield, D04 C1P1 Dublin, Ireland; caoimbhe.burke@ucdconnect.ie; 4 Life Sciences Institute, Dublin City University, D09 NR58 Dublin, Ireland; alex.eustace@dcu.ie; 5 School of Biochemistry and Cell Biology, Western Gateway Building, University College Cork, T12 XF62 Cork, Ireland; k.dean@ucc.ie; 6 School of Pharmacy and Biomolecular Sciences, RCSI University of Medicine and Health Sciences, D02 YN77 Dublin, Ireland; 7 UCD School of Medicine, UCD Conway Institute, University College Dublin, Belfield, D04 C1P1 Dublin, Ireland; arman.rahman@ucd.ie

**Keywords:** Irish Association for Cancer Research, early detection, targeted therapeutics, emerging hallmarks, artificial intelligence, data science, precision oncology

## Abstract

**Simple Summary:**

The Irish Association for Cancer Research (IACR) held its 59th annual conference from Wednesday, 22nd of February, to Friday, 24th February 2023, in Athlone, Ireland. The following article is a report of knowledge conveyed at the conference. The main themes of this conference explored key areas of basic, clinical, and translational research, which impact the diagnosis and treatment of cancer patients. The research presented at the conference emphasised the continuously changing paradigm within cancer research, with sessions covering a wide spectrum of topics from personalised prevention to personalised intervention, alongside emerging hallmarks, novel drug discoveries, and delivery systems. This was in conjunction with advancements in applications of artificial intelligence and data science within precision oncology that will ultimately revolutionise healthcare for cancer patients.

**Abstract:**

Advancements in oncology, especially with the era of precision oncology, is resulting in a paradigm shift in cancer care. Indeed, innovative technologies, such as artificial intelligence, are paving the way towards enhanced diagnosis, prevention, and personalised treatments as well as novel drug discoveries. Despite excellent progress, the emergence of resistant cancers has curtailed both the pace and extent to which we can advance. By combining both their understanding of the fundamental biological mechanisms and technological advancements such as artificial intelligence and data science, cancer researchers are now beginning to address this. Together, this will revolutionise cancer care, by enhancing molecular interventions that may aid cancer prevention, inform clinical decision making, and accelerate the development of novel therapeutic drugs. Here, we will discuss the advances and approaches in both artificial intelligence and precision oncology, presented at the 59th Irish Association for Cancer Research annual conference.

## 1. Introduction

Cancer is a leading cause of mortality worldwide. Currently, one in two men and one in three women will be diagnosed with cancer within their lifetimes. It is estimated that there were 18.1 million cancer cases accounting for almost 10 million deaths worldwide in 2020 [1]. Within Ireland, the incidence of cancer is over 42,000 cases per annum [2] and is expected to double again by 2050.

Alongside the overwhelming burden that cancer has on an individual’s life, there is a substantial economic impact worldwide. In 2023, it was reported that the estimated global economic cost of cancers from 2020 to 2050 will be USD 25.2 trillion, with distribution varying across cancer types, countries, and world regions [3]. These findings highlight that sustained global efforts are warranted to contain these economic predictions and to curb the ongoing burden of cancer.

The combination of artificial intelligence (AI) and precision medicine shows great promise in revolutionising healthcare in facilitating personalised diagnoses, prognoses, and treatment of patients by empowering clinical decision making through the toolbox of augmented intelligence. As described by Friedman’s fundamental theorem of informatics, the impact of augmented intelligence is that “the healthcare system with AI will be better than the healthcare system without it” [4,5]. Indeed, this will dramatically impact oncology, where there will be a paradigm shift in cancer care across the spectrum from enhanced prevention, diagnoses, and personalised treatments as well as novel drug discoveries [6].

**Figure 1:**
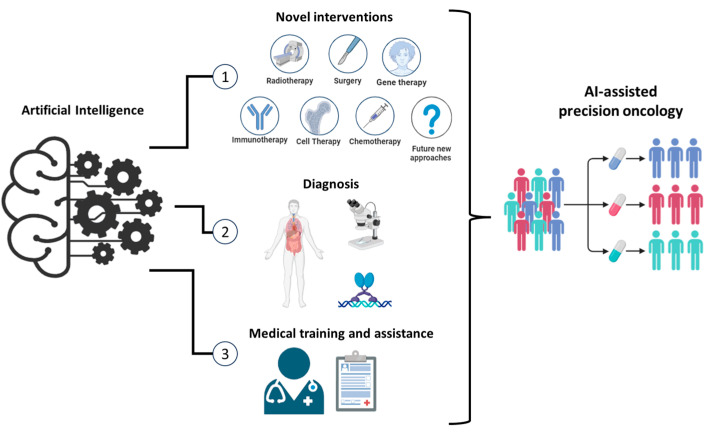
Transformative Potential of AI-Driven Precision Oncology. This figure presents an overview of the major themes and advancements discussed at the 59th IACR annual conference. It highlights the impact of AI-driven precision oncology across various domains, including novel approaches to treatment, diagnostic advancements, and enhancements in medical training and assistance. Novel Therapeutic Approaches: AI can accelerate the development of novel therapeutic approaches by integrating data from multiple omics, molecular profiling, and clinical trials. AI algorithms can identify potential drug targets, predict drug responses, and optimise combination therapies, leading to more effective and personalised cancer treatments. Advanced Diagnostic Techniques: AI can enhance diagnostic accuracy through advanced image analysis and pattern recognition. For example, machine learning models can analyse medical imaging data to detect cancerous lesions at early stages, often with greater precision than traditional methods. Medical Training and Clinical Decision Support: AI-driven platforms provide interactive training modules for medical professionals. Virtual reality and augmented reality technologies simulate complex surgical procedures and diagnostic scenarios, allowing trainees to practice and refine their skills in a risk-free environment. AI can also enhance clinical decision making by offering real-time, evidence-based recommendations. These systems integrate patient data, current medical research, and clinical guidelines to support oncologists in diagnosing and treating cancer. This reduces diagnostic errors and ensures that patients receive the most up-to-date personalised care.

At the 59th Irish Association for Cancer Research (IACR) conference, the sessions were reflective of this revolution, with speakers presenting on applications of AI and data science in precision oncology; emerging hallmarks of cancer, including senescence, drug persistence, and therapeutic vulnerabilities; molecular intervention from cancer prevention to treatment; and metastatic tumour models and novel drug delivery systems. Figure 1 provides an overview of the key themes discussed at the conference underscoring the transformative potential of AI-driven precision oncology and the collaborative efforts of researchers, clinicians, and industry stakeholders in advancing the frontiers of cancer diagnosis, prevention, and treatment.

## 2. Applications of AI and Data Science in Precision Oncology

Artificial intelligence (AI) and data science have emerged as indispensable tools in the realm of cancer research, extending far beyond personalised medicine and offering profound insights into the fundamental scientific understanding of complex biological processes [7]. At a molecular level, these technologies can unravel intricate interactions within biological systems, shedding light on crucial mechanisms such as gene expression regulation, protein interactions, and signal transduction pathways [8]. By analysing high-dimensional datasets comprising genomic, transcriptomic, proteomic, and epigenomic data, machine learning algorithms can identify subtle patterns and correlations that might evade traditional analytical approaches. This enables researchers to uncover novel biomarkers, pathways, and therapeutic targets that hold significant implications for advancing our understanding of diseases like cancer. Furthermore, these techniques facilitate the integration of diverse data modalities, allowing researchers to explore synergistic relationships and emergent properties within complex biological systems [9]. Ultimately, by harnessing the power of machine learning and data science, scientists can accelerate knowledge discovery and propel breakthroughs in biomedical research, paving the way for more effective diagnostic and therapeutic strategies in the fight against diseases.

Precision oncology, a field focused on tailoring cancer treatment to the unique characteristics of each patient, has seen significant advancements with the integration of AI and data science [10]. AI algorithms and data analytics play a crucial role in analysing vast amounts of genomic, clinical, imaging, and molecular data to provide personalised treatment strategies for cancer patients [11]. Additionally, AI and data science have been used to predict cancer treatment toxicity, enabling healthcare providers to develop personalised treatment plans that minimise side effects and optimise patient outcomes. Moreover, the use of AI and data science in precision oncology has also enhanced the process of cancer diagnosis, prognosis, and treatment selection [12]. Data captured from oncology providers and healthcare systems are complex and diverse. To gain valuable clinical insights and analytics, it is crucial to extract, process, analyse, interpret, and integrate relevant medical data in a proper and effective manner [13]. Increasing data availability and computing power have led to the development of advanced techniques such as machine learning and AI. These techniques are becoming increasingly important in addressing complex issues in cancer care.

Ms Aoife Ní Mhuirí, CEO and founder of Salaso Health Solutions, spoke on “The Role of Digital Therapy and AI in Precision Medicine” from an industry perspective, detailing its role in delivering software-generated therapeutic interventions directly to patients. This strategy not only aids in the prevention, management, or treatment of a medical disorder or disease, but also it supports clinical decision-making processes.

Using augmented intelligence and machine learning, Salaso developed Smart Therapy Engagement Platform and Services (STEPS), to advance digital transformation in the healthcare industry, enabling healthcare clients to develop new modalities of service delivery that meet their specific needs and are complementary to in-person care [14]. Harnessing the power of virtual care and care at home, healthcare providers can deliver effective evidence-based and clinically tested digital therapies as a service [15]. Patients are engaged to walk through their treatment journey on their own terms and timelines, with the freedom to complete in a place and at a time that suits them, while still being physician-led. Programmes designed using the platform have been used in multiple publications to deliver virtual physiotherapy and rehabilitation programmes in intestinal cancers [15], amongst others, to help mitigate side effects of treatment such as fatigue [16].

It is imperative to disseminate technologies such as AI and digital pathology on a global scale, as they have the potential to directly revolutionise healthcare systems. Johan Lundin, Professor of Medical Technology at the Global Public Health, Karolinska Institute, and Research Director at the Institute of Molecular Medicine Finland (FIMM), University of Helsinki, Finland, emphasised the significance of this transformation and the need for immediate action in places where it can have the maximum impact on patient care. He spoke on “AI and digital diagnostics for cervical cancer screening in resource-limited settings”. One of the most promising areas of AI in healthcare is its ability to enhance the visual interpretations made by clinicians, ultimately leading to improved patient outcomes. One such area is histopathology.

As it stands, the pathological diagnosis of the disease is reliant on the visual interpretation of images by a skilled expert. This is a proven bottleneck in terms of cancer screening and diagnosis, made even worse by a worldwide shortage of trained pathologists [17]. Inadequate access to pathology services is a major issue in low-income countries [18]. Shockingly, as highlighted by Professor Lundin, there can be less than one pathologist available per million people in these regions. This scarcity of expertise and resources has significant consequences for the health and well-being of millions of individuals. Urgent action is needed to address this critical public health challenge. Digital images of histology slides are an absolute necessity for AI-based algorithms to function, but unfortunately, the equipment required for digital pathology and cytology is not fit for point-of-care settings, and due to high cost, it is not feasible for resource-limited settings. Professor Lundin referred to one study of AI-based screening of cervical cancer in a catchment area of a rural hospital in Kenya using a prototype/portable slide scanner [19] and a cloud-based deep learning system to detect the cell atypia in Papanicolaou test (Pap smear test) samples [20].

Cervical cancer is the fourth most common cancer among women with 85% of diagnoses being in low- and middle-income countries [21]. The largest burden is in sub-Saharan Africa, where screening and vaccination programs have taken decades to implement [20]. Cancer diagnostic capabilities are at a critical level, with some countries having less than one pathologist per million on average for the manual evaluation of Pap smear slide samples [22]. From a study involving 740 patients, heralding from a rural clinic in Kenya, the sensitivity of the AI algorithm in detecting atypical cells in cervical smears was high (96–100%), which increased in high-grade lesions (93–99%) in comparison to low-grade lesions (82–86%) [19]. The algorithm identified 5% of samples to have low-grade atypia and 8% of the samples to have high-grade atypia. All women who were detected to have cellular changes by the AI algorithms received treatment such as cryotherapy at a hospital. In addition to these findings, no samples that were determined negative by a trained pathologist were incorrectly identified by the algorithm. Professor Lundin’s study clearly demonstrated that AI-based assistive technology-related innovations are likely to improve access to medical diagnostics and improve access to high-quality healthcare.

AI has been described as the third revolution in histopathology [23]. Although AI-based feature extraction has been widely used in Haematoxylin and Eosin (H&E) images to identify image-based cancer biomarkers, its use in multiplexed fluorescence immunohistochemistry (mIHC) is limited [24]. AI-powered feature recognition algorithms in commercial image analysis software enable the efficient detection of cells, precise tissue segmentation, and accurate identification of multiple markers within a sample. Since fluorescent signals are spectrally isolated, researchers can confidently identify phenotypes arising from low-expressing epitopes and count low phenotypic expressors among mixed cellular phenotypes. Dr Edwin Roger Parra Cuentas, from The University of Texas MD Anderson Cancer Center, spoke on immunoprofiling and the spatial mapping characterisation of non-small cell lung cancer (NSCLC) tissue samples using mIHC. This study analysed the expression of 23 markers placed in five mIHC panels, and different cell phenotypes were identified by marker co-expression. Digital pathology and image analysis were performed using PhenoImagerHT analyser (Akoya Biosciences, Marlborough, MA, USA).

The study found that patterns observed in the distance between different tumour-associated immune cells and malignant cells were associated with recurrence-free survival and overall survival [25]. Close distances of specific cellular neighbourhoods correlated with increased survival (CD3+, CD8+, CD45RO+, etc.) and decreased survival (CD3+, CD8-, FOXP3+, etc.) in NSCLC. Through studying the spatial distribution of different cell phenotypes in the tumour microenvironment, factors influencing immune surveillance, tumour progression, relapse, and overall survival could be elucidated [25].

## 3. Emerging Hallmarks of Cancer: Senescence, Drug-Tolerant Persisters, and Therapeutic Vulnerabilities

The landscape of cancer research is continually evolving, with new insights into the mechanisms driving tumourigenesis and therapeutic resistance. Traditional hallmarks of cancer, such as deregulated cellular metabolism, evasion of immune destruction, and genome instability, have been well studied. However, recent advancements have identified new critical aspects in cancer biology: cellular senescence, drug-tolerant persisters, and therapeutic vulnerabilities [26]. Understanding these factors is crucial for developing innovative and effective cancer treatments. Cellular senescence, a state of irreversible growth arrest, plays a dual role in cancer. While it can act as a barrier to tumourigenesis, preventing the proliferation of damaged cells, senescent cells can also create a pro-tumourigenic environment through the senescence-associated secretory phenotype. This complexity necessitates a nuanced approach to targeting senescence in cancer therapy. Drug-tolerant persisters are a subset of cancer cells that survive initial chemotherapy treatments by entering a reversible state of dormancy. These cells pose a significant challenge because they can re-enter the cell cycle and drive tumour recurrence once treatment ceases [27]. Targeting persisters before they acquire permanent resistance is essential for improving long-term treatment outcomes. While targeted therapies show promise initially in clinical trials, eventually therapeutic resistance occurs in late-stage disease [28,29]. Such resistance often arises from the reactivation of mitogenic signalling. Professor René Bernards of the Netherlands Cancer Institute has been investigating a paradoxical approach to cancer therapy. In his talk, “If you do what you did, you get what you got—changing the paradigm in cancer therapy”, Professor Bernards asked the question, “why do we continue to inhibit oncogenic signalling if the outcome is nearly always reactivation?” He discussed that natural selection within cancer cells that promotes the optimal, not necessarily the maximal, level of mitogenic signalling, and that the overactivation of these pathways may disrupt the homeostatic state of the cancer cells [30]. Evidence that synthetic lethality, through the potential overactivation of the mitogen-activated protein kinase pathway, underlies the mutual exclusivity of KRAS and EGFR oncogenic mutations in lung adenocarcinoma [31] supports this idea of homeostatic disruption through pathway overactivation.

Professor Bernards’s work investigates inhibiting protein phosphatase 2A (PP2A) to overactivate oncogenic signalling. PP2A is a serine/threonine phosphatase that has established tumour-suppressive functions [32,33,34]. While PP2A has traditionally been viewed as a tumour suppressor, more recent studies have demonstrated that the inhibition of PP2A has therapeutic potential. LB-100, a small-molecule inhibitor of PP2A, has shown efficacy in cancer models and is thought to work through the activation of mitogenic signalling [35]. Professor Bernards’s work initially investigated the effect of LB-100 on a panel of colorectal cancer (CRC) cell lines and demonstrated that LB-100 reduced cell proliferation across the panel of cell lines and activated oncogenic signalling. A synthetic lethality between Wee1-like protein kinase (WEE1) inhibition and PP2A inhibition was identified in two CRC cell lines. Combination treatment of LB-100 and the WEE1 inhibitor, adavosertib, exerted a synergistic anti-proliferative effect across in vitro models of CRC, pancreatic ductal adenocarcinoma, and cholangiocarcinoma [36].

Professor Bernards demonstrated that combination treatment with LB-100 and adavosertib led to mitotic catastrophe. Combination of LB-100 and adavosertib had an anti-tumour effect and lacked systemic toxicity in in vivo models. Finally, Professor Bernards demonstrated that acquired resistance to both PP2A and WEE1 inhibitions resulted in a reduced tumourigenic phenotype. When the resistant cells were implanted into in vivo models, tumours either failed to grow or small tumours developed [36].

Professor Bernards addressed the concern for the malignant transformation of normal cells. Normal cells possess more control mechanisms, which are frequently lost in cancer cells, to prevent uncontrolled proliferation [30]. Previous studies have shown that in vivo models overexpressing growth factors do not show increased tumourigenesis [37,38]. However, he acknowledged that extensive safety and toxicity testing would be required prior to the introduction of this therapeutic approach to the clinic, but nonetheless, this could be a useful therapeutic strategy in metastatic disease.

The theme of therapeutic resistance continued with a talk from Professor Catherine O’Brien from the University Health Network and Princess Margaret Cancer Centre, Toronto. Drug-tolerant persisters (DTPs) are slow-cycling cells that are reversibly resistant to chemotherapy, and upon the cessation of chemotherapy treatment, proliferate and grow. However, DTPs remain sensitive to the reintroduction of chemotherapy. These cancer cells are essentially capable of surviving exposure to chemotherapy without developing irreversible resistance [39]. Therefore, targeting DTPs prior to the development of irreversible drug resistance may be a useful therapeutic strategy.

Professor O’Brien’s talk entitled “Understanding the diapause-like drug tolerant persister state in CRC” discussed the similarities between DTPs and diapause, the reversible state of suspended embryonic development employed during periods of unfavourable environmental conditions [40,41]. Using patient-derived xenograft (PDX) models of CRC, Professor O’Brien’s group has demonstrated that DTPs maintain the genetic heterogeneity from the parent tumour, but irreversibly drug-resistant cells show a reduction in genetic heterogeneity, suggesting that DTPs have a non-genetic mechanism of drug tolerance. Using a high-complexity lentiviral barcode library, her group determined that DTPs did not represent a pre-existing subpopulation of cells within the tumour but that tumour cells exhibit an equipotency to become DTPs [42].

DTPs represent a reversible state. RNA sequencing (RNAseq) analysis showed that DTPs were distinct from vehicle-treated cells and irreversibly resistant cells, but cells that exited the DTP state and led to tumour regrowth were indistinguishable from vehicle-treated cells. A gene expression signature characteristic of embryonic pausing was generated by combining expression data from in vivo embryonic pausing with in vitro paused embryonic stem cells. The DTP transcriptome was found to mimic the embryonic diapause signature. DTPs are slow-cycling cells that show the upregulation of autophagy. Treatment with irinotecan in combination with autophagy inhibitors prevented DTP emergence [42].

Professor O’Brien demonstrated the clinical relevance of her findings through analysis of The Cancer Genome Atlas (TCGA) data of patients with CRC. Patients with a high diapause signature or a high autophagy signature had significantly poorer overall survival compared to patients that had a low diapause or a low autophagy signature. Professor O’Brien discussed that identifying patients with a high diapause or autophagy signature may identify patients likely to have resistant disease and allow for the development of novel therapeutic strategies to target these diapause-like DTPs [42].

The theme of therapeutic resistance continued with Professor Wilbert Zwart of the Netherlands Cancer Institute presenting, “Towards personalized therapy in hormone-driven cancers: from patients to models and back again”. Professor Zwart discussed his group’s work whereby they profiled epigenetically modified promoter/enhancer activities using histone H3, lysine 27 acetylation (H3K27ac) chromatin immunoprecipitation, and subsequent sequencing in samples from patients with metastatic castration-resistant prostate cancer (mCRPC). They identified 657 H3K27ac sites selectively enriched in patients who were resistant to androgen receptor-targeted therapy. This profile was successfully validated in PDX models of CRPC, whereby it was possible to classify the PDX tumours according to responders and non-responders based on the H3K27ac signal at the previously identified non-responder sites. Using cell line models, Professor Zwart’s group identified histone deacetylase 3 (HDAC3) as one of the key players in driving resistance to hormone-targeted therapies. Combined treatment with enzalutamide, an androgen receptor inhibitor, and vorinostat, a pan-HDAC inhibitor, demonstrated synergistic effects, therefore identifying a novel therapeutic strategy in mCRPC [43].

## 4. Molecular Intervention from Cancer Prevention to Treatment

Molecular intervention has become a cornerstone in the continuum of cancer management, spanning from prevention to treatment. This approach leverages insights into the molecular underpinnings of cancer to develop tailored strategies that optimise outcomes for patients. In cancer prevention, molecular interventions enable personalised risk assessment, early detection through biomarker identification, and targeted preventive measures. Meanwhile, in treatment, these interventions revolutionise therapy by targeting specific molecular aberrations within tumours, offering enhanced efficacy and reduced toxicity.

Enhanced prevention and treatment of cancer hinge upon a deeper understanding of the molecular mechanisms that drive cancer. Recent evidence has underscored the potential of “exercise as medicine” in improving clinical outcomes across various health conditions, including cancer. Exercise was reported to be associated with a decreased risk of 13 different cancers and may indeed increase survival after a cancer diagnosis [44]. Professor Bente Klarlund Pedersen from Rigshospitalet and University of Copenhagen, Denmark, has been investigating the molecular mechanisms linking exercise to cancer prevention and treatment. Professor Pedersen’s research group has shown that skeletal muscle cells are able to produce and secrete myokines [45]. The identification of myokines provides an insight into the molecular mechanisms of how muscles communicate with other organs, including cross-talk between muscle and liver, and muscle and fat [45]. The group has specifically identified the myokine prototype, interleukin-6 (IL-6), that is released from skeletal muscle during exercise. They found IL-6 to act as an energy sensor, as it enhances lipolysis and fat oxidation in humans in vivo. Furthermore, blocking endogenous IL-6 impaired the mobilisation of free fatty acids during rest and exercise in lean and obese men. Additionally, IL-6 enhanced insulin-stimulated glucose uptake in humans in vivo. These metabolic effects confirm that IL-6 works as an energy sensor [45,46].

Skeletal muscle behaving as an endocrine organ during exercise is a discovery that has led to a new theory that there will be chaotic malfunction of other organs and tissues in the body if the endocrine function of the muscle is not stimulated through exercise. Previous studies focused on investigating the effect of exercise training on tumour growth in mice have revealed marked reductions in tumour incidence and growth with voluntary wheel running across several different tumour models. The mechanism underlying this was demonstrated to be mediated through the direct regulation of natural killer (NK) cell mobilisation and trafficking in an epinephrine- and IL-6-dependent manner to ultimately control tumour growth [47]. Further research identified catecholamines to be involved in activating the Hippo tumour suppressor signalling pathway in the exercise-dependent suppression of breast cancer [48]. Exercise was also shown to mediate anti-inflammatory effects, and the link between physical inactivity, accumulation of visceral fat and IL-6 has been investigated. Indeed, IL-6 was reported to be required for exercise-mediated reduction in adipose tissue and chronic inflammation [49,50]. These results collectively support the anti-tumourigenic effects of IL-6, which are elevated after exercise.

The coupled action of IL-6 and epinephrine in the blood resulted in the increased mobilisation of NK cells that migrate into tumours and destroy tumour cells. Exercise training appears to prepare the tumour environment for the action of these cells by enhancing the expression of ligands for receptors of NK cells [49,50]. Further evidence of exercise as medicine was demonstrated in patients with gastroesophageal junction cancer, where exercise training was found to increase the number of patients reaching tumour resection, reduce the number of patients who are hospitalised during neoadjuvant chemotherapy, and reduce the toxicities resulting from neoadjuvant chemotherapy [51].

Professor Pedersen concluded by referring to the group’s ongoing study, the PRESET Phase-3 Randomised Controlled Trial. Patients (*n* = 210) with stage I-III gastroesophageal adenocarcinoma will enrol into treatment arms with exercise (two weekly sessions) versus standard of care with the primary endpoints of failure to return to intended surgery, 3-year overall and disease-free survival. Professor Pedersen’s translational research focus from this work is to develop targeted exercise training regimes for specific disease groups by applying a translational strategy: “from bedside to bench and back”.

The next talk in this session was delivered by Professor Ruth Travis, Professor of Epidemiology and Senior Molecular Epidemiologist, University of Oxford. The focus of this talk was the plasma proteome and prostate cancer risk, including insights from genetic and prospective analyses. Professor Travis highlighted that the identification of genetic determinants of circulating proteins in large-scale consortium studies offers unique opportunities to assess the relevance of thousands of proteins in cancer aetiology through Mendelian randomisation techniques in a cost-efficient manner. This will ultimately help our understanding of risk factors and inform strategies for prevention. Professor Travis highlighted the clinical need to identify risk factors for aggressive prostate cancer. The strategic approach to address this unmet need entails the global collaboration of large, detailed studies, including aggressive prostate cancer, interdisciplinary research networks, and the triangulation of results from different types of studies. Furthermore, the identification of blood proteins linked to cancer will inform about disease risk factors, biological pathways for disease, biomarkers for screening and early detection, and potential therapeutic prevention targets. This information can come from blood measurements, genetic studies, and single and multiplex protein analyses. Various resources already exist including the prospective cohort data from the EPIC-Prostate: European Prospective Investigation of Cancer [52], the UK Biobank [53], and other prospective cohorts worldwide, alongside other resources including the genetics consortia SCALLOP [54] and PRACTICAL [55], and this means that the data can be collated to give high power. Indeed, using this strategic approach, insulin-like growth factor-1 (IGF-1) was identified as a single protein biomarker, with higher IGF-I concentration associated with increased risks of thyroid, colorectal, breast, and prostate cancer and reduced risk of ovarian and liver cancer based on prospective analyses in the UK Biobank [56,57]. Studies have shown evidence that this biomarker has a protective role in prostate cancer development [58]. Professor Travis highlighted ongoing work using the EPIC-Prostate resource to analyse single plasma proteins, kallikreins, and multiplex protein analyses, utilising OLINK antibody and next-generation sequencing-based panels. This approach takes advantage of new technology, both in terms of “omics” and electronic data linkage. Professor Travis’s take-home message was that large-scale studies on a collaborative level can be used to determine risk, enhance screening and early detection, determine prognosis, and ultimately inform treatment strategies for cancer patients.

The final talk in this plenary session welcomed Dr Philip Dunne, Reader in Molecular Pathology, Patrick Johnston Centre for Cancer Research, Queen’s University Belfast and Group Leader at the Cancer Research UK (CRUK) Beatson Institute in Glasgow. The focus of this talk was the reactivation of anti-tumour immune surveillance to prevent disease progression in CRC. Dr Dunne’s translational research program primarily focused on changing the paradigm in cancer therapy for patients with early-stage CRC. Dr Dunne set the scene for this talk by alluding to the findings from the NICHE-2 clinical trial of patients with mismatch repair-deficient (dMMR) colon cancer as reported at the European Society for Medical Oncology (ESMO) Congress 2022. Data from this clinical trial showed, for the first time, a major pathological response in 95% of patients within 6 weeks of receiving neoadjuvant immunotherapy. The findings highlight that the introduction of such neoadjuvant immunotherapy as standard of care in early colon cancer has the potential to be paradigm changing [59]. Dr Dunne suggested that we could use biology-driven transcriptional biomarkers in this space for patients with early-stage CRC.

Molecular and transcriptional subtyping has identified the worst prognostic group in early-stage CRC as stroma-rich tumours, with high transforming growth factor-beta (TGFβ), as these tumours respond the least to standard of care chemotherapy [60,61,62]. Additionally, targeting of TGFβ provides no clinical efficacy [63]. Therefore, treatment for such stroma-rich tumours needs to change, and Dr Dunne has identified a new trial platform that gives a potential window of opportunity. Stroma-rich tumours can be easily and rapidly identified [64,65]. It was reported that the low expression of the HiFi-prognostic signature (HPS), alongside the low expression of signal transducer and activator of transcription 1 (STAT1) and interferon (IFN) signalling, was associated with a high relapse rate. More specifically, the high expression of HPS within tumours was linked to high nuclear factor kappa B (NFκB) and interferon regulatory factors/signal transducer and activator of transcription (IRF/STAT) transcription factor levels. The biology underpinning this was targeted by a repurposing approach using polyinosinic polycytidylic acid. Indeed, targeting of the biology driving this response using the polyinosinic polycytidylic acid (poly(I:C)) approach was found to reduce metastatic burden in a stroma-rich model of CRC [65]. Further research is ongoing to further elucidate the mechanism of action behind this poly(I:C) response with two phase II neoadjuvant trials in development. Dr Dunne highlighted that this represents an opportunity to improve outcomes in the worst prognostic group for patients with CRC through the identification, mechanistic interrogation, and therapeutic targeting of the tumour–immune–stromal interactions. These interactions underpin disease relapse in these patients, and Dr Dunne’s work displayed the need to facilitate the translation of mechanistic science into clinical trials.

## 5. Metastatic Tumour Models and Novel Drug Delivery Systems

In the closing plenary session of the conference, metastatic cancers took centre stage, alongside the emergence of novel drug delivery systems. This pivotal session underscored the clinical significance of innovative drug delivery approaches that will shape the treatment landscape for metastatic tumours.

Metastatic cancers pose formidable challenges due to their advanced stage and propensity to spread to distant sites in the body. Conventional treatment modalities often face limitations in effectively targeting metastatic lesions while minimizing systemic toxicity. However, novel drug delivery systems offer promising solutions by enhancing the specificity, efficacy, and safety of therapeutic interventions.

Opening the final plenary session was Professor David Wink from the National Institute of Health (NIH). Professor Wink delivered a talk centred on nitric oxide synthase-2 (NOS2) and cyclooxygenase-2 (COX2) as major drivers of poor prognosis of oestrogen receptor-negative (ER-) breast cancer, through shaping the immune landscape and tumour microenvironment. Professor Wink suggested that NOS2 and COX2 may be used to overcome immunosuppression and pitched the talk around this central theme. Nitric oxide synthase (NOS) and cyclooxygenase (COX) were previously implicated in the redox landscape during inflammation [66]. Indeed, studies have mapped nitric oxide and nitrosation as activators of key oncogenic pathways, with NOS2 noted as a predictor of poor clinical outcome in different cancers [67,68,69]. Within the breast cancer setting, intrinsic inflammatory loops in the tumour microenvironment drive breast cancer with elevated levels of NOS2 and COX2 noted to be collaborating in a feedforward loop [70]. Furthermore, NOS2 has been reported to drive both metastasis and chemoresistance [71,72,73,74]. The stimulus for this induction is linked to a combination of proinflammatory cytokines, such as interferon gamma (IFNγ) and tumour necrosis factor alpha (TNFα). The IFNγ and cytokines provide the maximal expression of NOS2 and COX2 in ER- breast cancer cells with IFNγ, Th1, IL-1, TLR4, TNF, and Th17 cytokines associated with high NOS2 and high COX2 levels. The tumour IFNγ is secreted from lymphoid cells, and there is a dichotomy of IFNγ and Th1 cytokines promoting NOS2/COX2 signalling yet being required for tumour eradication and associated with improved clinical outcomes [67,71,72,73,74].

A translational 3D platform for understanding the tumour ecosystem in aggressive ER-, triple-negative breast cancer (TNBC) and human epidermal growth factor receptor 2-positive (HER2+) breast cancer was utilised to investigate if there was a spatial relationship between the lymphoid cells and NOS2/COX2. Single-cell intensity with fluorescence was used as this provides single-cell analysis of the tumour microenvironment. NOS2 and COX2’s single-cell intensity was found to correlate with survival, with proximity of CD8+ cells and IFNγ correlating with NOS2 expression. Further analysis using heat density maps revealed the progression of inflammation to immune desert regions in the same tumour. Indeed, regions with the high expression of NOS2 were shown to result in increased satellitosis and metastatic potential, resulting in cancer stem-like and metastatic niches associated with poor prognoses. Ultimately, Professor Wink reported that spatial analysis of the tumour microenvironment including NOS2 and COX2’s location impacts the immune distribution in the tumour microenvironment [75]. To investigate if NOS2 and COX2 were novel targets for the treatment of ER- breast cancer, further studies were performed to investigate this potential mechanism. Pharmacological treatment of NOS2 and COX2 with the inhibitor indomethacin resulted in reduced tumour growth and complete tumour regression in an in vivo model of breast cancer [76]. Moreover, it was further reported that the NOS2- and indomethacin-augmented response is reflected in systemic differences in the immune system [76]. Professor Wink ended his talk by emphasising that targeting NOS2 and COX2 systemically leads to dramatic positive change in the immune response that can augment conventional and immune therapies. The results show promise of a novel immunotherapeutic approach for treatment of aggressive tumours including TNBC.

Continuing with the theme of metastatic cancers and novel drug delivery systems, Professor Richard J. Gilbertson from the University of Cambridge presented his talk entitled, “Will we ever cure cancer?” Professor Gilbertson’s research is focused on understanding the link between normal development and the origins of cancer, particularly in brain tumours. In his talk, Professor Gilbertson asked the question, “Does development set the ground state for all cancers?” He discussed the main hypothesis of cancer origins, including the theory that cancer development can be driven by “bad luck” as the result of random mutations in proliferating stem cells [77]. Evidence in the scientific literature suggests that developing and ageing tissues may be described like funambulists with sufficient cell plasticity to prevent malignant transformation. Therefore, rather than considering “bad luck”, Professor Gilbertson explained how deciphering the complex orchestration of the cellular mechanisms that induce transformation represents a promising avenue to prevent, detect, and cure cancer [78]. He explained that cancer stem cells (CSCs) are very much like normal stem cells, as they are multipotent, they can renew, and they present common surface markers, such as PROMININ-1 (Prom-1). What is not fully elucidated is if CSC are the descendants of mutated stem cells, thus answering whether normal stem cells are susceptible to cancer.

Professor Gilbertson demonstrated that Prom-1 marked the stem cells in the small intestine of adult *Prom1^+^/C-L* mice, and that activation of endogenous Wnt signalling increased the *Prom1^+^* stem cell population, leading to malignant transformation of the mucosa of the small intestine in the mice [79]. These data demonstrated that cancerous cells in this model have a portion of transformed stem cells. Furthermore, Professor Gilbertson discussed how *Prom1^+^*-associated stem cell capacity and cancer risk varies in space and time. Different organs have different risks to undergo malignant transformation, and different types of tumours occur in children and adults. This spatial and temporal variation in cancer transformation can be related to the organ-specific ability to react to carcinogens and oncogenic mutations [80], but more studies are needed to better understand the mechanism regulating tumourigenesis, in order to ameliorate the prevention and treatment of cancer. Using a series of conditional oncogene and tumour-suppressive gene alleles, mutations were introduced into well-characterised stem and non-stem cell populations across different organs in neonatal (postnatal day [P]1) and adult (P60) mice, to test the role of intrinsic factors in developing cancer. Professor Gilbertson demonstrated that the risk of a specific organ to develop cancer is significantly associated with the life-long generative capacity of the mutated cells, regardless of their developmental stage, strongly supporting the role of stem cells in cancer risk development [81]. This model also showed that cellular damage induced by extrinsic factors can induce the acquisition of stem cell properties by relatively plastic cell populations. In addition, stem cells can also regulate the temporal sequence of cancer risk in the same organ: adult *Prom1^+^* cells are more likely to undergo cancerous transformation when compared to neonatal *Prom1^+^* cells with equivalent generative capacities [81]. These findings may explain why cancer rates are significantly lower in children than adults, even though childhood malignancies accumulate high numbers of non-synonymous mutations [82].

In his talk, Professor Gilbertson also discussed how the development of anti-cancer therapies to treat metastatic tumours is difficult, due to the limited knowledge of targets in primary tumours driving metastasis [83]. Interestingly, Professor Gilbertson identified the sodium leak channel non-selective protein (NALCN) as a key cellular regulator of the metastatic process [84]. He showed how the control of NALCN function represents a promising new avenue to prevent cancer metastasis. NALCN deletion had no impact on key primary tumour characteristics and did not alter tumour incidence, but significantly increased the number of circulating tumour cells and metastases of gastric, intestinal, and pancreatic adenocarcinomas in mice. Interestingly, gadolinium contrast agent, a NALCN channel blocker, markedly accelerated cancer metastases by increasing circulating recombined tumour-derived cells. Professor Gilbertson also showed that NALCN deletion may regulate epithelial cell shedding, by upregulating the expression of genes linked to epithelial–mesenchymal transition (EMT) and invasion. Deletion of NALCN in mice that lacked oncogenic mutations caused shedding of epithelial cells into the blood at levels equivalent to those seen in tumour-bearing animals, demonstrating that NALCN governs cell shedding from tissues independent of cancer, therefore representing an oncogene-independent metastatic pathway that can be targeted for anti-metastatic therapies [84]. Professor Gilbertson concluded his talk by highlighting the fact that we might get to the point where we can prevent cancer by understanding the minimal requirements for tumour development, what is missing from repairing adult stem cells, and discovery of targets to prevent metastasis.

For the final speaker of this plenary session, the IACR welcomed Professor William Jacot from Montpellier University, who was an investigator on the DESTINY-04 clinical trial. This trial resulted in the approval of the HER2–antibody drug conjugate trastuzumab deruxtecan in patients with HER2 low-metastatic breast cancers [85,86]. Professor Jacot explained how HER2 overexpression at either the gene or protein level has long been associated with a poorer patient outcome. However, HER2 itself can be a target for drug treatment. Professor Jacot reviewed the importance of HER2-oncogenic addiction and how this has been exploited as a treatment strategy using antibodies such as trastuzumab and pertuzumab [87]. The results of these studies have led to improved outcomes for women with advanced HER2-positive breast cancer [88].

Professor Jacot outlined that HER2 itself could be used as a receptor to help target the delivery of chemotherapy directly to the cancer cell using antibody–drug conjugates (ADCs). ADCs such as trastuzumab emtansine (TDM-1) use the antibody trastuzumab to target the cancer cell. Using a linker system, the antibody can then be directly conjugated to a chemotherapy drug. When trastuzumab binds to the HER2 protein, it is internalised by endosomal cycling. A reduction in pH in the lysosome results in the linker being degraded, enabling the release of the chemotherapy and killing of the cancer cell. Results from the EMILIA clinical trial demonstrated the benefit of this approach, and TDM-1 was approved for use in metastatic HER2-positive breast cancer [89]. Indeed, recent results from the KATHERINE studies have shown its benefit in the adjuvant treatment of HER2-positive breast cancer [90]. Next-generation HER2-ADCs including T-DXd, which have more advanced linker systems, and different payloads, have also been developed [91]. These have also been approved for the treatment of HER2-positive breast cancers through the DESTINY-03 clinical trial [92].

Importantly, HER2 expression (3+ by immunohistochemistry) is variable across breast cancer patients and can be classified as HER2 2+ or 1+. These patients are not classified as HER2-positive and have lower expression of the HER2 protein (HER2-Low), but Professor Jacot stated that this population of HER2-low breast cancers may also benefit from the use of HER2-ADCs such as T-DXd. The DESTINY-04 clinical trial was established to test this hypothesis and included only patients with HER2-low breast cancer including those with ER+ and TNBC [85]. Results of this trial have recently demonstrated that T-DXd improved both relapse-free survival and overall survival in this population of metastatic breast cancers. Professor Jacot ended this plenary session by highlighting that the results of this trial mean that HER2-targeted drugs such as ADCs can now be tested in a wider population of breast cancers, increasing the number of women who can benefit from this drug.

## 6. Award Sessions

The Irish Cancer Society guest speaker, Professor Peter J. O’Dwyer, Professor of Medicine, University of Pennsylvania, delivered an outstanding talk on complementary technological and conceptual advances in cancer research. Professor O’Dwyer referred to a multitude of studies that included genomic analyses, immunotherapeutics, real-world data, real-world evidence, and multi-cancer testing. The first element alluded to during his talk was that genomic analysis, including next-generation sequencing, has given rise to a plethora of genomically driven clinical trials, including the landmark TAILORx clinical trial for breast cancer patients [93,94,95,96,97] and the precision medicine cancer clinical trial NCI-MATCH [98,99]. Underpinning genomically driven clinical trials is the key considerations in the molecular triage trial design, including the hypothetical framework for a genomically driven trial, the tumour biopsy type, the biomarker platform, and the treatment settings. However, the lack of therapeutic success seen is due to drugs not being good enough, drugs applied too late in the disease course, the presence of co-occurring mutations that confer resistance, and single agents, which were never going to be effective drugs. Professor O’Dwyer indicated that the existing trials should be utilised to identify targets and guide therapy in the direction of the most promising therapeutic avenues and ultimately give rise to better drugs.

The talk then moved into the era of immunotherapeutics and precision immune therapy as a viable concept. The broadening role of immunotherapeutics and major advances in antibody-targeting antibody–drug conjugates, bispecific antibodies, and Bispecific T-Cell Engagers (BiTES) are paving the way for integration of such modalities in the future [85,100,101]. The phase III randomised trial, E1910, was used an illustrative example where the overall survival advantage was evident in newly diagnosed B-lineage acute lymphoblastic leukaemia patients who received blinatumomab combined with chemotherapy who were minimal residual disease (MRD) negative after intensification chemotherapy [102]. Similar to these findings is the results of the DESTINY-Breast04 trial, where Enhertu, a HER2-directed ADC, was found to significantly improve both progression-free and overall survival in HER2-low expressing breast cancer patients, highlighting that BiTEs may be relevant for patients at the limits of biological detection [85].

Professor O’Dwyer highlighted that real-world data must be utilised to accelerate and guide drug development in clinical trials [103]. However, challenges do exist in collecting such real-world data, especially given that only 3% of patients access clinical trials within the USA. Some of the challenges include that data can be collected from Phase IV trials, but this is not enough by itself, since target populations are underrepresented in all clinical trials. In addition, many obstacles in mining electronic health records exist including defining response, estimating when progression occurred, and full documentation of toxicity. Thus, to approach and overcome such challenges, technological solutions such as AI and natural language processing are critical in order to reimagine the infrastructure of cancer to design and operate better clinical trials, accelerate drug development, and increase access, which will ultimately power smarter care for all cancer patients. Since a small percentage of patients go on clinical trials, this means that learning is extremely limited. The goal should be increased to 30% of patients enrolling on to clinical trials. This will provide a valuable opportunity to permit the collection of real-world data that informs our future cancer care and therapy.

The final element of Professor O’Dwyer’s lecture focused on multicancer early detection (MCED) testing and study design considerations for trials of MCED, with the goal of decreasing cancer death rates. Indeed, early detection is the rationale for all screening, but risk, benefit, and cost effectiveness need to be established [104]. Two clinical studies have been developed in this space that demonstrate the feasibility of using such MCED tests: Detecting cancers Earlier Through Elective mutation-based blood Collection and Testing (DETECT-A) [105] and GRAIL [106]. The DETECT-A study, which is based on >10,000 women with no known cancer history, uses circulating tumour DNA (ctDNA) and protein markers, combined with positron emission tomography–computed tomography (PET-CT), to screen for cancer and guide intervention. Results from this study demonstrated that blood testing can detect early cancer in those without any history of the disease, whilst presenting as asymptomatic. Thus, this testing can be relied on to direct intervention, primarily surgery with intent to cure. The results from this study highlighted that multi-cancer blood testing combined with PET-CT can be safely incorporated into routine clinical care [105]. Moreover, the GRAIL methylation profiling study also confronts fundamental questions around the use of MCED testing and provides the clinical validation of a targeted methylation-based MCED test. This test showed high specificity and accuracy of cancer signal origin prediction and detected cancer signals across a wide diversity of cancers [106]. Professor O’ Dwyer highlighted that MCED testing will decrease cancer death rates, and this will remain essential for government-funded research, with the need for public funding to focus on prevention, early detection, expansion of technology, and evaluation of testing effectiveness. Ultimately, MCED testing will complement and assist clinicians in clinical decision making for all patients. Professor O’Dwyer in the finishing remarks alluded to the Cancer Moonshot initiative that launched in 2016 [107]. The fundamental goal of this initiative is to accelerate discovery, foster greater collaboration and expansion of cancer data sharing among the research community, reduce the cancer death rate by half within 25 years, and improve the lives of people with cancer and cancer survivors.

The conference concluded with the final speaker and awardee of the IACR Award for Outstanding Contribution to Cancer Medicine and Research, Professor Elaine Kay, Consultant Histopathologist, Royal College of Surgeons Ireland/Beaumont, Ireland. Professor Kay’s talk, “Coming Full Circle: The Evolution of Pathology in Human Disease Research”, explored the historical progression and modern advancements in the field of pathology. She began by defining pathology as the study of disease, both in the medical sense and as extreme and unreasonable behaviour.

Professor Kay delved into the historic writings on pathology, tracing back to Hippocrates in 400 BC, who made significant contributions to anatomy and pathology through the first dissections of deceased humans. It also covered the concept of diseases being “organ” based, as advocated by Morgagni in the 1600s [108]. The introduction of microscopy in the 1800s revolutionised pathology, allowing for the examination of tissues with greater detail. This led to the emergence of histopathologists, who play a vital role in diagnosing diseases based on cellular changes. Rudolf Virchow’s work on cellular pathology, including his famous aphorism “omnis cellula e cellula” (“every cell stems from another cell”), further advanced the field.

The talk highlighted the significance of staining techniques such as H&E, which have stood the test of time, as standard stains for histological examination whilst also touching upon the variability in pathology diagnosis, as demonstrated by studies on observer accuracy and reproducibility [109]. Advancements in staining techniques, including special stains like Ziehl–Neelsen and immunohistochemistry (IHC), have expanded the diagnostic capabilities of pathology. Molecular pathology, particularly the use of techniques like fluorescent in situ hybridisation (FISH) and molecular profiling, has ushered in an era of personalised medicine, enabling targeted therapies based on genetic mutations [110].

Professor Kay also discussed the challenges and variations in the pathology diagnostic pathway, emphasizing the importance of specimen sampling, staining, and reporting accuracy. The advent of digital pathology, facilitated by technologies like whole-slide imaging and AI, has further transformed the field, enhancing efficiency, accuracy, and collaboration among researchers [111,112]. In conclusion, this final talk of the 59th IACR conference by Professor Kay provided a comprehensive overview of the evolution of pathology, from its historical roots to modern technological advancements, highlighting its critical role in understanding and combating human diseases such as cancer.

## 7. The IACR 60th Anniversary Special Symposium

In 2024, the European Association for Cancer Research (EACR) and American Association for Cancer Research (AACR) in partnership with the IACR organised the excellent EACR-AACR-IACR 2024 Basic and Translational Research Conference [113], which took place in Dublin, Ireland, on 27–29 February 2024. Indeed, 2024 marks the 60th anniversary of the IACR. To mark this special event, the IACR held a special sitting at this international meeting, the “IACR 60th Anniversary Special Symposium” as the preceding event of this prestigious international meeting. This meeting highlighted the latest cutting-edge advances in the development of cancer and response to therapy from preclinical models to patients.

## 8. Discussion and Concluding Remarks

The landscape of oncology is undergoing a profound transformation propelled by advancements in precision oncology, novel therapeutic strategies, and the integration of AI technologies. Precision oncology, characterised by the molecular profiling of tumours and the identification of vulnerable therapeutic targets, heralds a paradigm shift in cancer management. By leveraging genomic, transcriptomic, and proteomic data, clinicians can decipher the underlying molecular drivers of cancer and tailor treatment strategies to individual patients. Targeted therapies, immunotherapies, and molecularly guided clinical trials offer unprecedented opportunities for improved outcomes and reduced toxicity in oncology practice. Artificial intelligence, particularly machine learning and deep learning algorithms, is poised to transform cancer care by augmenting diagnostic accuracy, prognostic assessment, and therapeutic decision making. AI-powered tools analyse vast datasets, including medical imaging, genomic sequencing, and electronic health records, to identify patterns, predict treatment responses, and stratify patients based on risk profiles. From image-based tumour detection and classification to predicting drug responses and patient outcomes, AI algorithms offer invaluable insights that complement traditional diagnostic and prognostic approaches. Despite remarkable progress, the emergence of resistant cancers poses a formidable challenge to oncology advancement. Resistance mechanisms, encompassing genetic mutations, tumour heterogeneity, and microenvironmental interactions, thwart treatment efficacy and limit therapeutic options. To overcome this hurdle, cancer researchers are leveraging their understanding of fundamental biological mechanisms in tandem with AI and data science tools. Integrating multi-omic data, longitudinal patient records, and real-time monitoring, AI-driven approaches enable the identification of predictive biomarkers, elucidation of resistance mechanisms, and optimisation of treatment regimens to circumvent resistance and improve patient outcomes.

The convergence of precision oncology and artificial intelligence holds promise for revolutionising cancer care. By synergistically combining molecular insights with computational power, researchers and clinicians can unlock new avenues for cancer prevention, early detection, and treatment optimisation. Molecular interventions informed by precision oncology data and guided by AI algorithms have the potential to mitigate resistance, tailor therapies to individual patients, and accelerate the development of novel therapeutic agents. As precision oncology and AI continue to advance, several challenges and opportunities lie ahead. Standardizing data collection, ensuring data privacy and security, validating AI algorithms in diverse patient populations, and integrating AI tools into clinical workflows represent key priorities. Moreover, interdisciplinary collaboration between oncologists, data scientists, computational biologists, and bioinformaticians is essential for harnessing the full potential of these technologies and translating scientific discoveries into tangible clinical benefits.

The 59th IACR annual conference showcased excellent research and crosslinked various themes within basic, clinical, and translational cancer research, which impacts on the diagnosis and treatment of patients. The integration of precision oncology and artificial intelligence heralds a new era of cancer care characterised by personalised, data-driven approaches. By harnessing the collective power of molecular insights and computational algorithms, we are poised to overcome the challenges of resistant cancers and usher in transformative advancements in cancer prevention, diagnosis, and treatment.

Importantly, at the 59th IACR conference, there was a strong emphasis on Patient and Public Involvement (PPI) in cancer research. A PPI workshop coordinated by Ms. Kay McKeon (IACR PPI Council Champion) and an IACR PPI stand, hosted by patient advocates, were held during the conference to raise the profile and awareness of PPI in Cancer Research in Ireland, to increase PPI participation in the IACR Conference, and to ensure that researchers have opportunities to engage with PPI partners throughout the research cycle.

## Data Availability

Not applicable.

## Abbreviations

ADCs	antibody–drug conjugates
AI	artificial intelligence
AACR	American Association for Cancer Research
BiTES	Bispecific T-Cell Engagers
COX	cyclooxygenase
COX2	cyclooxygenase-2
CRC	colorectal cancer
CRPC	castration-resistant prostate cancer
CRUK	Cancer Research UK
CSC	cancer stem cells
ctDNA	circulating tumour DNA
dMMR	mismatch repair-deficient
DTP	drug-tolerant persister
EACR	European Association for Cancer Research
EMT	epithelial–mesenchymal transition
ER	oestrogen receptor
FIMM	Institute of Molecular Medicine Finland
FISH	fluorescent in situ hybridisation
H&E	Haematoxylin and Eosin
HDAC	histone deacetylase
HER2	human epidermal growth factor receptor 2
HPS	HiFi prognostic signature
IACR	Irish Association for Cancer Research
IL-6	interleukin-6
IGF-1	insulin-like growth factor-1
IFN	interferon
IFNγ	interferon gamma
IHC	immunohistochemistry
IRF	interferon regulatory factors
MCED	multicancer early detection
mIHC	multiplexed fluorescence immunohistochemistry
MRD	minimal residual disease
MSP	microseminoprotein-beta
NALCN	sodium leak channel non-selective protein
NIH	National Institute of Health
NFκB	nuclear factor kappa B
NK	natural killer
NOS	nitric oxide synthase
NOS2	nitric oxide synthase-2
NSCLC	non-small cell lung cancer
PDX	patient-derived xenograft
PP2A	protein phosphatase 2 A
PPI	Patient and Public Involvement
STAT	signal transducer and activator of transcription
STAT1	signal transducer and activator of transcription 1
STEPS	Smart Therapy Engagement Platform and Services
TDM-1	trastuzumab emtansine
T-DXd	trastuzumab deruxtecan
TGFβ	transforming growth factor-beta
TNFα	tumour necrosis factor alpha
TNBC	triple-negative breast cancer
